# Component communities and annual and seasonal variations of metazoan parasites in *Eleotris pisonis* (Gmelin, 1789) (Gobiiformes: Eleotridae) in the Amazon River, Brazil

**DOI:** 10.1590/S1984-29612023073

**Published:** 2023-12-04

**Authors:** Elvis Silva Lima, Marcos Tavares-Dias

**Affiliations:** 1 Programa de Pós-graduação em Biodiversidade e Biotecnologia (Rede Bionorte), Universidade Federal do Amapá – UNIFAP, Macapá, AP, Brasil; 2 Universidade do Estado do Amapá – UEAP, Macapá, AP, Brasil; 3 Embrapa Amapá, Macapá, AP, Brasil

**Keywords:** Aggregation, freshwater fish, infection parasites, seasonality, Agregação, peixe de água doce, infecção por parasitos, sazonalidade

## Abstract

Our hypothesis for this study was that annual and seasonal variations do not influence the structure of the component communities and the diversity of metazoan parasites of spinycheek sleeper (*Eleotris pisonis*) in the Amazon River, state of Amapá, Brazil. A total of 164 fish were collected between 2020 and 2021, from which 888 parasites were found. In 2020, five species of parasites were found (one Nematoda, one Digenea, one Acanthocephala, one Arachnida and one Crustacea); and in 2021, five species were also found (three Nematoda, one Digenea and one Crustacea). Larvae of *Contracaecum* sp. were the dominant taxon throughout the study. The parasite species richness and Brillouin diversity index were higher in 2021, without significant differences between seasonal periods. Some component communities of parasites showed differences between years and between seasonal periods. These facts do not support the hypothesis that such variables would not influence the component communities of the parasites. Lastly, this report provides the first records of *Spirocamallanus inopinatus*, *Genarchella genarchella*, Acari, *Ergasilus* sp., *Neoechinorhynchus* sp., larvae of *Pseudoproleptus* sp. and larvae of *Contracaecum* sp. in *E. pisonis*.

## Resumo

Suponha-se que variações anuais e sazonais não influenciavam a estrutura das comunidades componentes e a diversidade dos parasitos metazoários de peixes, Amoré Preto, *Eleotris pisonis* do Rio Amazonas, estado do Amapá (Brasil). Um total de 164 peixes foram coletados de 2020 a 2021 e 888 parasitos foram encontrados nos peixes estudados. Em 2020, foram encontradas cinco espécies de parasitos (um Nematoda, um Digenea, um Acanthocephala, um Arachnida e um Crustacea); e, em 2021, também foram encontradas cinco espécies três Nematoda, um Digenea e um Crustacea). Larvas de *Contracaecum* sp. foram o táxon dominante ao longo do estudo. A riqueza de espécies de parasitos e o índice de diversidade de Brillouin foram maiores em 2021, enquanto entre as estações sazonais não houve diferenças significativas. Algumas comunidades componentes de parasitos apresentaram diferenças entre os anos e entre os períodos sazonais. Esses fatos não corroboram a hipótese de que tais variáveis não influenciariam as comunidades componentes dos parasitos. Por fim, este é o primeiro registro de *Spirocamallanus inopinatus*, *Genarchella genarchella*, Acari, *Ergasilus* sp., *Neoechinorhynchus* sp., larvas de *Pseudoproleptus* sp. e larvas de *Contracaecum* sp. para *E. pisonis*.

## Introduction

The spinycheek sleeper *Eleotris pisonis* (Gmelin, 1789) (Gobiiformes: Eleotridae) is widely distributed along the western margin of the central Atlantic Ocean, from Bermuda, Bahamas, South Carolina and the northern Gulf of Mexico in the United States at the northern end of its range to Brazil at the southern end of its range (Froese & Pauly, 2023). Spinycheek sleepers prefer freshwater estuarine tributaries with a salinity range from 0 to 36.6 psu (Darcy, 1980; Ray & Robins, 2016), and adult individuals are found in shallow water with a muddy or sandy bottom (Cervigón, 1994). They are carnivorous fish that feed on dipteran larvae and pupae, small crustaceans such as crabs and shrimps, and small fish. In addition, cannibalism has also been reported, but with low frequency (Perrone & Vieira, 1991). Their diet varies according to their state of sexual maturity and with the seasons. They are small-sized fish that reach sexual maturity at lengths of 5.7 cm for males and 4.3 cm for females. Spawning occurs in the dry season (Nordlie, 1981; Planquette et al., 2000; Santos et al., 2004). However, studies on the communities and infracommunities of metazoan parasites in this fish species are scarce.

The parasite communities of freshwater fish are important components of biodiversity, as they provide information about their environments such as water quality. In addition, they influence the productivity and food web of ecosystems (Negreiros et al., 2019a; Lehun et al., 2022). Therefore, studies on wild fish parasite communities can generate information on how these parasite-host-environment relationships can respond to ecological actions such as host diet, environmental characteristics, existence of infective stages in the ecosystem and annual and seasonal variations (Negreiros et al., 2019a; Hoshino & Tavares-Dias, 2019; Hoshino & Tavares-Dias, 2020; Lima et al., 2021, 2022, 2023; Lehun et al., 2022), and also the strategies used by different taxa of parasites in relation to these variables.

In temperate climate regions, it is known that water temperature and the behavior of wild fish populations play important roles in the dynamics of infection caused by parasites (Schade et al., 2016; Yang et al., 2016). Many aquatic invertebrates in these regions are potential intermediate, paratenic or definitive hosts for fish. These characteristics contrast with those of tropical regions, where aquatic ecosystems do not have extreme temperatures, which can fluctuate during the year.

Fish can harbor ectoparasites and endoparasites of different species with different life cycles. The species of parasites harbored are often related to the behavior and diet of the host. In this way, species occupying different niches are exposed to different parasites, thus potentially resulting in different patterns of infection (Tavares-Dias et al., 2014; Tavares-Dias & Oliveira, 2017; Baia et al., 2018; Cavalcante et al., 2020; Lima et al., 2022). Studies have shown that the rainy and dry seasons can influence the behavior of host fish, as well as the diversity of parasites and invertebrates in ecosystems. Thus, identifying the factors that can influence the structure of parasite communities is important for better understanding of the parasite ecology of host fish.

Abiotic and biotic factors in the environment may be related to temporal and seasonal variations in the structure of parasite communities in wild fish populations. Studies have shown that wild fish in the Amazon basin may present variations in their parasite communities according to temporal and seasonal periods (Hoshino & Tavares-Dias, 2019; Negreiros et al., 2019a; Hoshino & Tavares-Dias, 2020; Lima et al., 2023).

The tropical climate of the eastern Amazon region is mainly influenced by the Amazon rainforest, where the rainy season occurs from December to May and the dry season from June to November (Souza & Cunha, 2010). Rainfall levels influence the physicochemical characteristics of aquatic ecosystems during seasonal periods, consequently influencing fish populations and parasite communities. However, information on the effects of seasonality on the dynamics of infections in wild fish populations in the Amazon is scarce (Negreiros et al., 2019a; Hoshino & Tavares-Dias, 2020; Lima et al., 2022, 2023). For example, there are no studies on annual and seasonal variations in the communities of metazoan parasites in *E. pisonis*.

Organisms can respond similarly to abiotic and biotic factors, resulting in parallel patterns in community structures across taxonomic groups, called community concordance (Jackson & Harvey, 1993). However, temperature is an example of a variable that can influence both the immune system of host fish populations and the reproduction rate of parasites: innate immunity is more active at low temperatures, while adaptive immunity is suppressed at low temperatures (Ondračková et al., 2015). Thus, in temperate climate regions, seasonal variations in temperature can alter not only fish metabolism but also parasite communities (Ondračková et al., 2015; Rohlenová et al., 2011). In tropical climate regions, communities are expected to have a stable structure throughout the year (Dias & Tavares‐Dias, 2015). Thus, the hypothesis of the present study was that annual and seasonal variations do not influence the parasite communities of *E. pisonis*. The aim of this study was to characterize the community of metazoan parasites in *E. pisonis* in the Amazon River and to study the effects of annual and seasonal variations on the structure of their parasite communities.

## Materials and Methods

### Study area and fish collection

**Figure 1:**
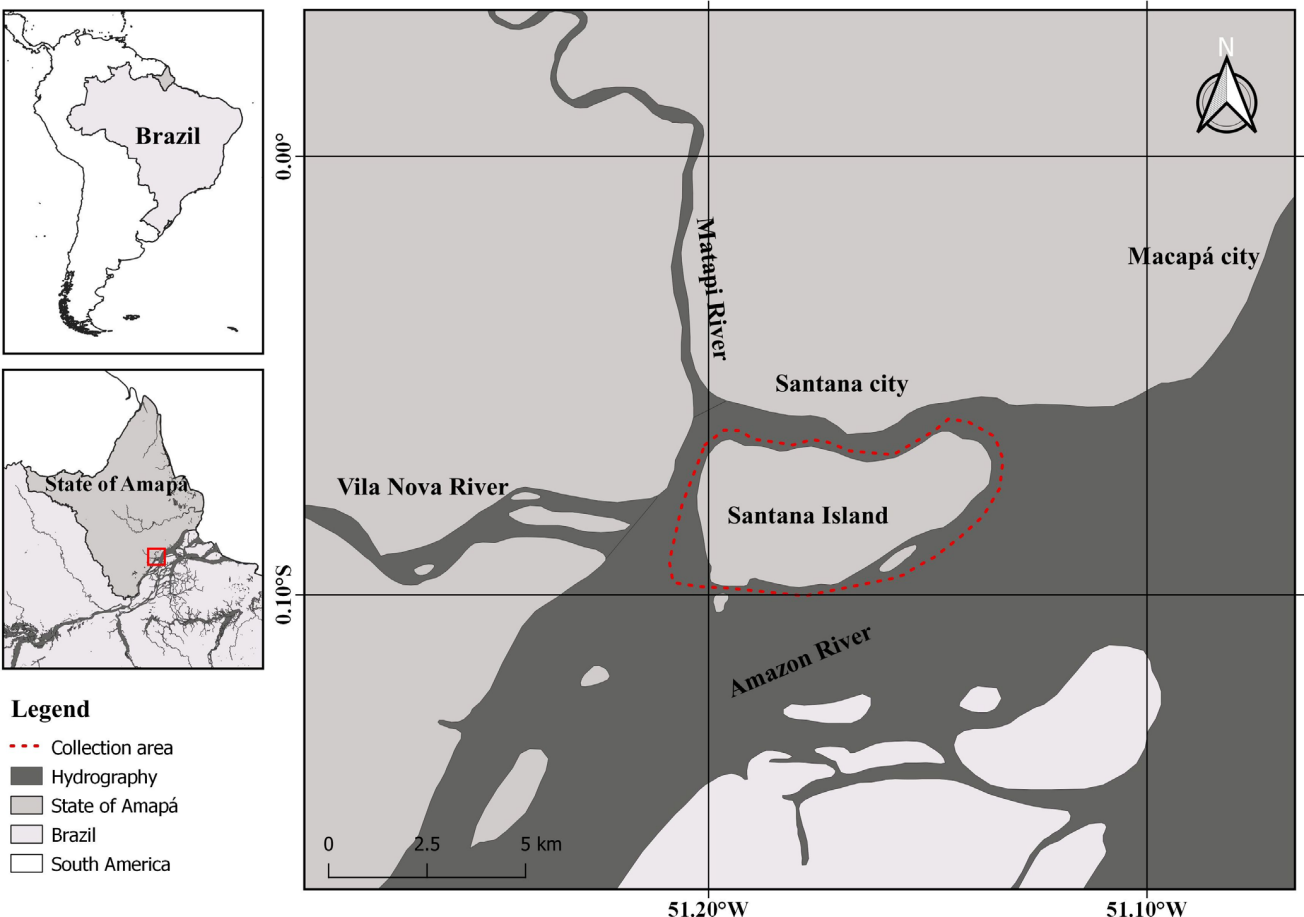
Collection site of *Eleotris pisonis* in the Amazon River, state of Amapá, northern Brazil.

In bimonthly collections from January 2020 to November 2021, a total of 164 specimens of *E. pisonis* [7.77 ± 1.77 cm (4.1-12.2 cm) and 7.98 ± 6.10 g (0.8-38.9 g)] were collected from the Amazon River, near to Santana Island, municipality of Santana, state of Amapá, Brazil (Figure 1). These fish were collected using gillnets of different sizes and meshes (15, 20, 25, 30 and 35 mm between knots), cast nets (20 mm mesh between knots) and hand lines. The fish were then sacrificed using the medullary transection method, preserved in 10% formalin and transported to the Aquaculture and Fisheries Laboratory of Embrapa Amapá, Macapá, state of Amapá, Brazil, in order to analyze their parasites.

The water quality parameters of electrical conductivity, pH and total dissolved solids were measured at the fish sampling sites using a multiparameter device (AKSO, model COMBO5-02-1016). Dissolved oxygen levels and water temperature were measured using an oximeter (Instrutherm, model MO-900). Rainfall data from the fish collection region were obtained from the Center for Hydrometeorology and Renewable Energy (NHMET) of the Institute of Scientific and Technological Research of the State of Amapá (IEPA).

### Parasite analysis procedures

In the laboratory, the total length (cm) and weight (g) of the fish were measured, and necropsies were performed to collect metazoan parasites. The mouth, opercular cavity, gills and fins were examined with the aid of a stereomicroscope to collect ectoparasites, while internal organs such as the gastrointestinal tract and viscera were examined for endoparasites. The parasites found were preserved in 70% ethyl alcohol in order to subsequently make permanent slides, following previous recommendations (Eiras et al., 2006).

### Data analysis

The parasite indices of prevalence, mean intensity and mean abundance of infracommunities were calculated (Bush et al., 1997). The dispersion index (DI) was determined and the DI significance was calculated using *d*-statistics (Ludwig et al., 1988). The Poulin discrepancy index (D) was calculated using the Quantitative Parasitology 3.0 software. To describe the parasite community, the species richness, Brillouin diversity index (*HB*), evenness (*E*) and Berger-Parker dominance index (*d*) were calculated using the Diversity software. The alternation of parametric and non-parametric tests was done in accordance with the normality tests for each set of data analyzed here. Spearman's correlation coefficient (*rs*) was used to evaluate possible correlations of host body length and weight with parasite abundance, species richness and Brillouin diversity index (Zar, 2010).

Fish weights and lengths were used to determine the relative condition factor (Kn) (Le Cren, 1951) for each year (2020 and 2021), for both the rainy and the dry season. To determine whether the total length (cm), weight (g), relative condition factor (Kn), physical-chemical parameters, prevalence, abundance, species richness, uniformity, Brillouin diversity index and Berger-Parker dominance index followed patterns of normal distribution and homoscedasticity, the Shapiro-Wilk and Bartlett tests were used, respectively. To ascertain whether there was any difference in the prevalence of parasites between 2020 and 2021, and between the seasonal periods (rainy and dry), the chi-square test (χ^2^) with Yates correction was used. To determine any differences in the abundance of parasites, the Mann-Whitney test (*U*) was used (Zar, 2010). To estimate any differences in diversity indices (species richness, evenness, Brillouin diversity index and Berger-Parker dominance index) among the metazoan parasites between 2020 and 2021 and between seasonal periods, the Kruskal-Wallis test was used, followed by the Dunn test. These analyses were carried out in the R software (R Core Team, 2021).

Permutation Analysis of Variance (PERMANOVA) was used to detect any differences in the parasite communities between the years 2020 and 2021 and between the rainy and dry seasons. Annual and seasonal variables were adjusted by means of principal coordinate analysis (PCoA) ordering based on the Bray-Curtis distance, using the envfit function from the vegan package (Oksanen et al., 2020), and p-values were calculated using the permutation test (number of permutations = 999) in the R software, version 2.5-2, using the vegan package (Oksanen et al., 2020).

To analyze how differences in sampling effort might influence the results, we plotted species accumulation curves (observed and expected) for 2020 and 2021 and for the rainy and dry seasons. To compare the diversity between the years studied and between the rainy and dry seasons, diversity profiles were generated based on Hill numbers (Hill, 1973), which on a q scale incorporates species richness (q = 0), exponential value (q = 1) and the inverse of the Shannon-Weaver and Simpson indices (q = 2). An increase in the q scale represents an increase in the weight given to common species in the diversity indices (Magurran & McGill, 2011), thus making it possible to identify how diversity is ordered and whether changes occur in relation to dominant or rare species (Tóthmérész, 1995). The diversity profile was built using the 'renyi' function from the vegan package (Oksanen et al., 2020). These analyses were carried out in the R software (R Core Team, 2021).

## Results

### Physicochemical parameters in the years 2020 and 2021 and seasonal periods

The pH and total dissolved solids measured in the years 2020 and 2021 showed significant differences (t = 2.4242, p < 0.05; *U* = 2.0, p < 0.05), but the other parameters analyzed did not show any significant differences between the years (Table 1). Between the seasonal periods (rainy and dry), there were significant differences in precipitation and temperature, respectively (t = 3.9908, p< 0.001; t = 6.8690, p < 0.001), but the other parameters analyzed did not show any significant differences (Table 1).

**Table 1:** Physicochemical parameters of water from the Amazon River, eastern Amazon region, Brazil, during periods of collection of *Eleotris pisonis*.

			**Tests**	
**Parameters**	**2020**	**2021**	***t***	***U***
Rainfall (mm)	195.7 ± 119.5	231.5 ± 153.2	-0.8197	-
Temperature (°C)	29.0 ± 1.0	29.1 ± 1.1	0.0284	-
Dissolved oxygen (mg/L)	5.8 ± 0.2	4.2 ± 1.8	2.2699	-
pH	7.1 ± 0.2	6.8 ± 0.3	2.4242*	-
Total dissolved solids (mg/L)	56.8 ± 10.7	107 ± 60.7	-	2.0^*^
Electrical conductivity (µS/cm)	90.4 ± 12.2	148.1 ± 111.4	-	10.5
	**Rainy season**	**Dry season**		
Rainfall (mm)	311.5 ± 106.7	131.0 ± 116.3	3.9908^**^	-
Temperature (°C)	28.3 ± 0.3	30.0 ± 0.5	6.8690**	-
Dissolved oxygen (mg/L)	5.2 ± 0.8	4.6 ± 2.12	0.4952	-
pH	6.9 ± 0.2	6.9 ± 0.3	-0.2389	-
Total dissolved solids (mg/L)	72.6 ± 34.6	91.4 ± 62.5	-	16.00
Electrical conductivity (µS/cm)	98.8 ± 61.4	139.6 ± 98.7	-	15.50

*t:* t test; *U:* Mann-Whitney test;

* p < 0.05;

** p < 0.001.

### Component communities of metazoan parasites

*Eleotris pisonis* was found to be parasitized by larvae of *Contracaecum* Railliet & Henry, 1912; *Pseudoproleptus* Khera, 1953 and *Spirocamallanus inopinatus* Travassos, Artigas & Pereira, 1928 (Nematoda); *Genarchella genarchella* Travassos, Artigas & Pereira, 1928 (Digenea); *Neoechinorhynchus* Stiles & Hassall, 1905 (Acanthocephala); *Ergasilus* Nordman, 1832 (Ergasilidae); and Acari Krantz, 1978 (Arachnida) (Voucher: 171P-177P-IEPA). *Contracaecum* sp. was the dominant species, and no parasite was found in the mouth or fins of the hosts (Table 2).

**Table 2:** Spatial distribution of metazoan parasites of *Eleotris pisonis* in the Amazon River, eastern Amazon region, Brazil.

**Parasite species**	**P (%)**	**MA ± SD**	**MI ± SD**	**TNP**	**FD (%)**	**SI**
**Nematoda**						
*Contracaecum* sp. (larvae)	7.9	0.1± 0.5	0.6 ± 1.1	22	2.4	Intestine
*Contracaecum* sp. (larvae)	48.7	2.1 ± 4.8	0.2 ± 6.3	354	39.8	Stomach
*Contracaecum* sp. (larvae)	28.0	0.9 ± 2.1	0.3 ± 2.9	147	16.5	Abdominal cavity
*Contracaecum* sp. (larvae)	0.6	0.01 ± 0.08	1 ± 0	1	0.1	Liver
*Pseudoproleptus* sp. (larvae)	0.6	0.01 ± 0.08	1 ± 0	1	0.1	Intestine
*Spirocamallanus inopinatus*	0.6	0.01 ± 0.08	1 ± 0	1	0.1	Abdominal cavity
**Digenea**						
*Genarchella genarchella*	21.9	0.9 ± 2.5	0.2 ± 3.8	155	17.4	Pharynx
*Genarchella genarchella*	10.9	0.2 ± 1.3	0.4 ± 3.3	45	5.0	Intestine
*Genarchella genarchella*	16.4	0.5 ± 1.5	0.3 ± 2.6	84	9.4	Stomach
*Genarchella genarchella*	1.8	0.06 ± 0.5	0.3 ± 2.5	10	1.1	Abdominal cavity
*Genarchella genarchella*	0.6	0.04 ± 0.4	0.1 ± 0	6	0.6	Pyloric cecum
**Acanthocephala**						
*Neoechinorhynchus* sp.	0.6	0.01 ± 0.08	1.0 ± 0	1	0.1	Intestine
*Neoechinorhynchus* sp.	1.2	0.02 ± 0.17	0.6 ± 0.7	3	0.3	Stomach
**Crustacea**						
*Ergasilus* sp.	17.0	0.3 ± 1.4	0.4 ± 3.06	57	6.4	Gills
**Arachnida**						
Acarina gen. sp.	0.6	0.01 ± 0.08	1.0 ± 0	1	0.1	Gills

P: prevalence; MA: mean abundance; MI: mean intensity; TNP: total number of parasites; FD: frequency of dominance; SI: sites of infection; SD: standard deviation.

The parasite community showed low species richness, low HB and predominance of endoparasites (Table 3). Only *Contracaecum* sp., *G. genarchella* and *Ergasilus* sp. presented prevalence above 10%, and these were analyzed separately. The hosts were predominantly infected by only one species of parasite. Larvae of *Contracaecum* sp. (DI= 4.83, *d* = 21.59 and D = 0.87), *G. genarchella* (DI = 5.03, *d* = 22.49 and D = 0.74) and *Ergasilus* sp. (DI = 2.07, *d* = 6.77 and D = 0.87) showed highly aggregated distribution patterns.

**Table 3:** Component community of metazoan parasites in *Eleotris pisonis* from the Amazon River, state of Amapá, in Brazil.

Paramenters	Values
Number of fish examined	164
Total number of parasites	888
Total prevalence (%) of parasites	82.3
Percentage of endoparasites (%)	93.4
Percentage of ectoparasites (%)	6.5
Percentage of larvae	59.1
Species richness of parasites	1.3 ± 0.9
Brillouin diversity index	0.2 ± 0.2
Evenness	0.8 ± 0.4

Species richness (*rs* = 0.28, p = 0.0004 and *rs* = 0.28, p = 0.0004), Brillouin diversity index (*rs* = 0.25, p = 0.0013 and *rs* = 0.24, p = 0.0013), abundance of *Contracaecum* sp. (*rs* = 0.46, p ≤ 0.0001 and *rs* = 0.47, p ≤ 0.0001), abundance of *Ergasilus* sp. (*rs* = 0.27, p = 0.0003 and *rs* = 0.29, p = 0.0001) showed weak but significant positive correlations with host length and weight, respectively.

### Annual variation of metazoan parasites

Host fish collected in 2020 had a mean length of 8.0 ± 1.8 cm and those collected in 2021 had a mean length of 7.5 ± 1.6 cm, which was a significant difference (*t* = 2.02, p = 0.04). Fish collected in 2020 had a mean weight of 9.2 ± 7.1 g and those collected in 2021 had a mean weight of 6.8 ± 4.7 g, which was also a significant difference (*U* = 2745.5, p = 0.04). The relative condition factor (Kn) of hosts collected in 2020 (Kn = 1.02 ± 0.39) and 2021 (Kn = 0.85 ± 0.67) were significantly different (*U* = 2434.0, p = 0.0013).

**Figure 2:**
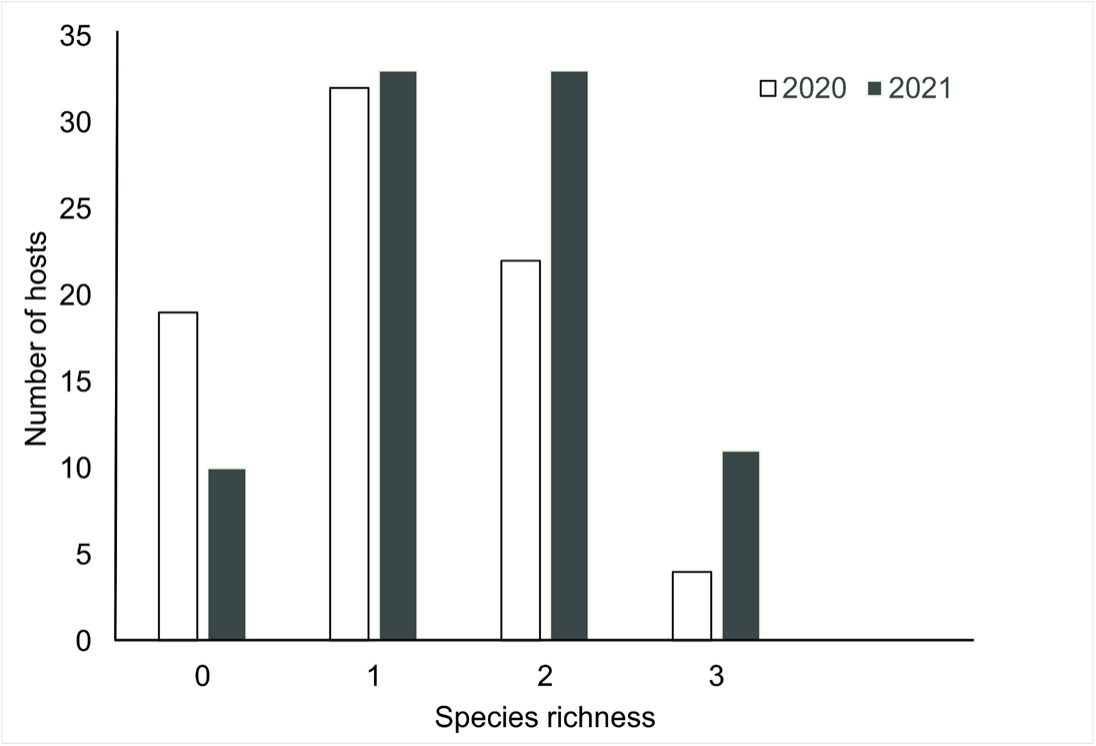
Species richness of metazoan parasites in *Eleotris pisonis* collected from the Amazon River, Brazil, over a two-year period.

Among all the hosts examined, a total of 368 parasites were recovered in 2020, while 520 were recovered in 2021. In 2020, hosts were predominantly infected by one parasite species, while in 2021 they were infected by one or two parasite species (Figure 2). In 2020, the total prevalence of parasites was 75.3% and in 2021 it was 88.5%. In both years, *Contracaecum* sp. was the dominant parasite, with higher prevalence in 2021. However, between these two years, there were significant differences in the prevalence and mean abundance of *G. genarchella* (Table 4).

**Table 4:** Metazoan parasites in *Eleotris pisonis* in the Amazon River, eastern Amazon region, Brazil, collected over a two-year period.

		**2020 (n = 77)**			**2021 (n = 87)**				
**Parasite species**	**Infection sites**	**P (%)**	**MA ± SD**	**TNP**	**P (%)**	**MA ± SD**	**TNP**	**χ^2^**	***U***
*Contracaecum* sp. (larvae)	Intestine, stomach abdominal cavity and liver	63.6	3.6 ± 6.9	281	70.1	2.7 ± 3.6	243	0.7	3200.0
*Pseudoproleptus* sp. (larvae)	Intestine	0	0	0	1.1	0.01 ± 0.1	1	-	-
*Spirocamallanus inopinatus*	Abdominal cavity	0	0	0	1.1	0.01± 0.1	1	-	-
*Genarchella genarchella*	Pharynx, intestine, stomach, abdominal cavity and cecum pyloric	24.6	0.6 ± 1.7	47	65.5	2.9 ± 3.7	253	27.4^**^	1804.5**
*Neoechinorhynchus* sp.	Intestine and stomach	3.9	0.05 ± 0.2	4	0	0	0	-	-
Acarina gen. sp.	Gills	1.3	0.01 ± 0.11	1	0	0	0	-	-
*Ergasilus* sp.	Gills	24.6	0.4 ± 1.9	35	13.7	0.2 ± 0.7	22	0.3	3142.5

P: prevalence; MA: mean abundance; SD: standard deviation; TNP: total number of parasites; χ^2^: chi-square test; U: Mann-Whitney test;

** p < 0.001.

**Figure 3:**
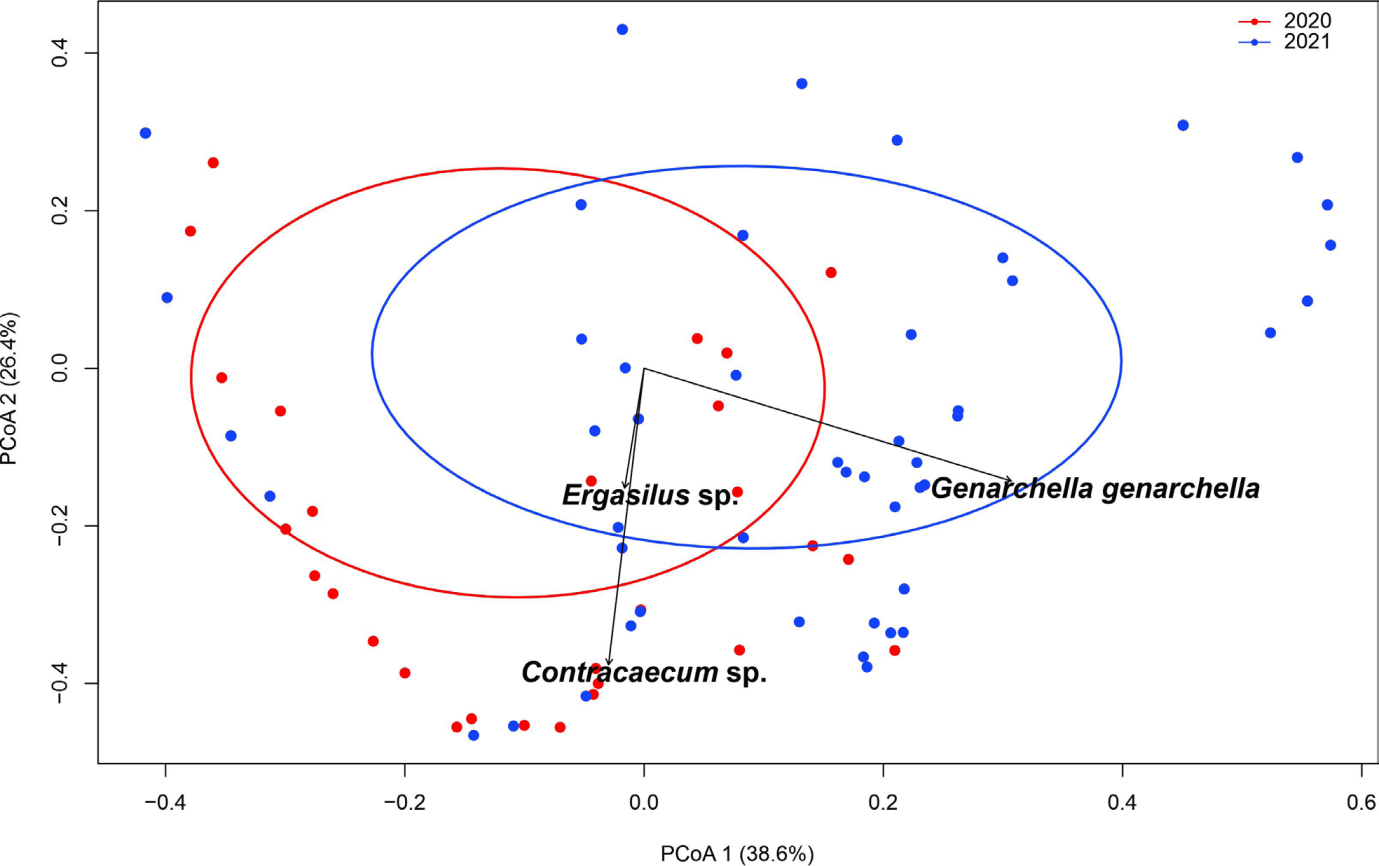
Principal coordinate analysis (PCoA) using a Bray-Curtis distance matrix for communities of metazoan parasites of *Eleotris pisonis* in the Amazon River, state of Amapá, Brazil, during 2020 and 2021. The percentage of the variation explained by the principal coordinates plotted is indicated on the axes.

The PCoA axes 1 and 2 were responsible for 65% of the total variation in abundance composition in 2020 and 2021. Although the PCoA showed overlapping and species sharing during these years, there were significant differences (PERMANOVA: F = 7.7445, p ≤ 0.001) in the parasite infracommunities, influenced mainly by variations in the abundance of *Contracaecum* sp. (R^2^ = 0.4275, p ≤ 0.001), *G. genarchella* (R^2^ = 0.3443, p ≤ 0.001) and *Ergasilus* sp. (R^2^ = 0.0701, p = 0.002) (Figure 3).

**Figure 4:**
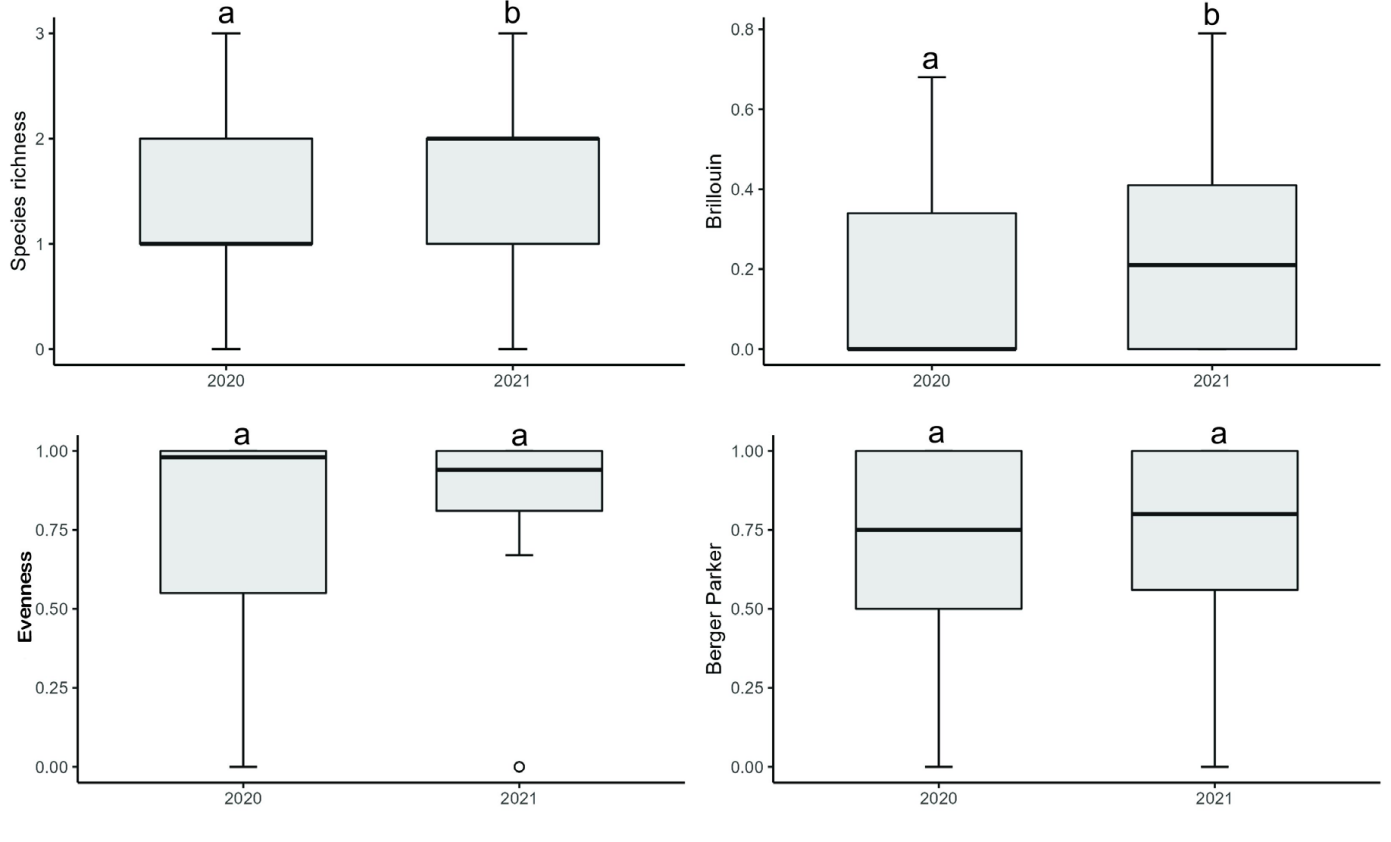
Diversity parameters for metazoan parasites in *Eleotris pisonis* in the Amazon River, eastern Amazon region, Brazil, collected in 2020 and 2021. (Box plots show medians, interquartile ranges, minimum-maximum ranges and outliers. Different letters indicate differences between the medians according to Dunn's test (p < 0.001).

**Figure 5:**
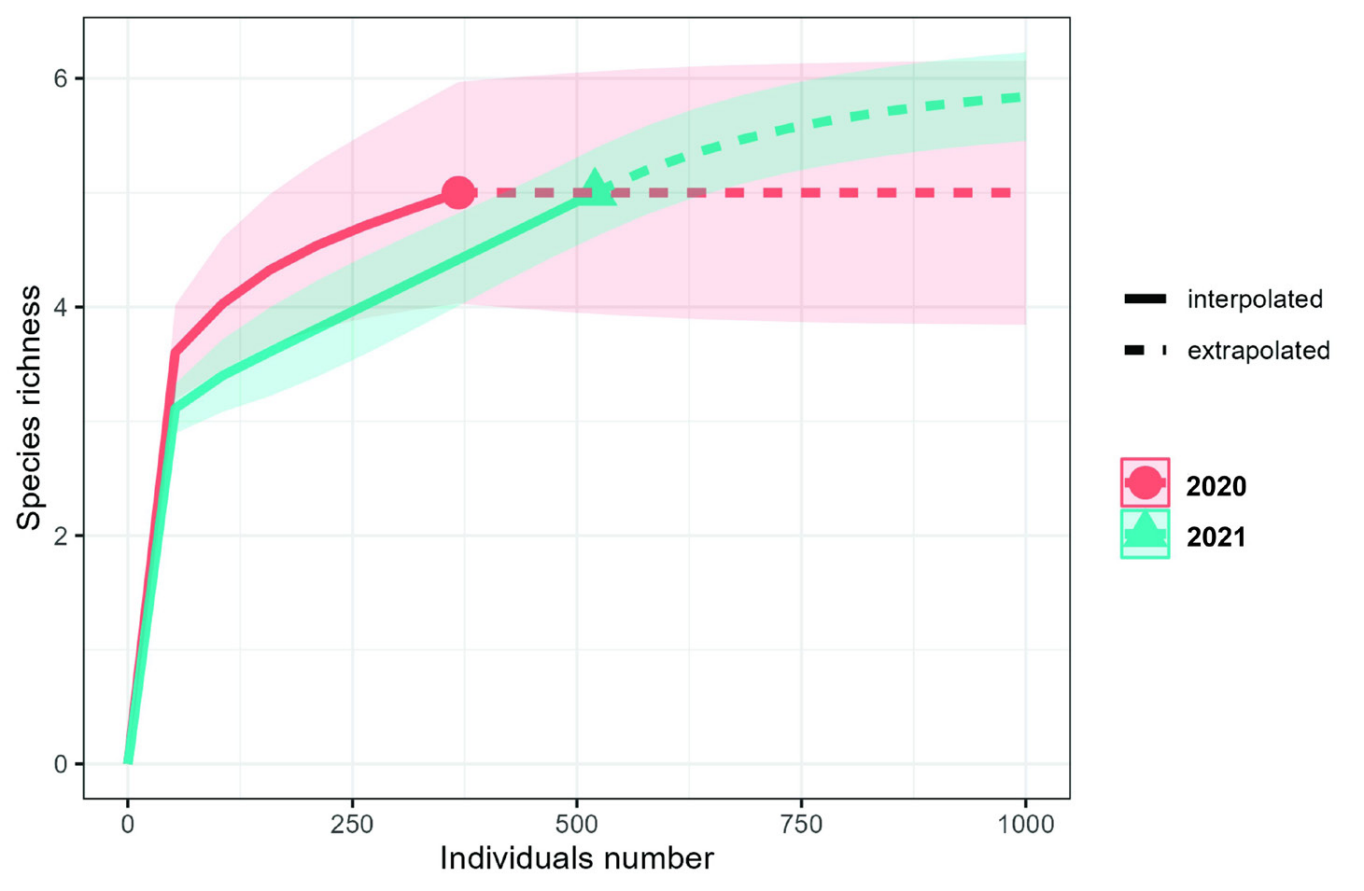
Species accumulation curve for metazoan parasites in *Eleotris pisonis* in the Amazon River, state of Amapá, Brazil, collected in 2020 and 2021.

Parasite species richness (χ^2^ = 7.23, p = 0.0071) and Brillouin diversity index (χ^2^ = 4.6729, p = 0.0306) were higher in 2021, but evenness (χ^2^ = 0.0069, p = 0.9335) and Berger-Parker dominance index (χ^2^ = 0.5514, p = 0.4577) showed no differences between the years studied (Figure 4). The species accumulation curve showed that the number of hosts found in 2020 was sufficient for the number of parasite species collected to reach representativeness, however, the 2021 species accumulation curve did not demonstrate a tendency towards stability, not being enough so that the number of parasite species collected would reach representativeness, after collecting the samples (Figure 5).

**Figure 6:**
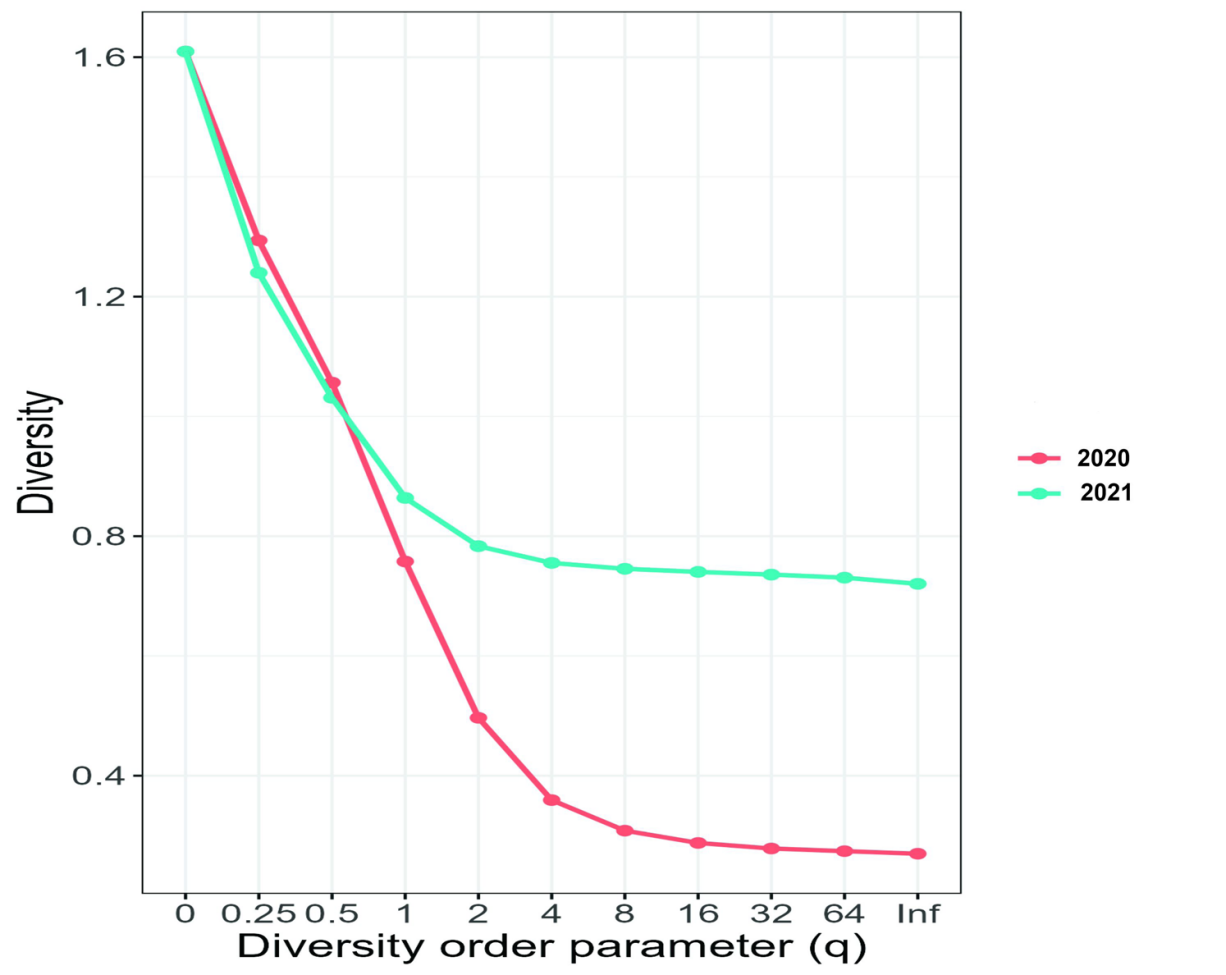
Hill diversity profile for diversity of parasites in *Eleotris pisonis* in 2020 and 2021. On the horizontal axis (left) rare species become more important, while towards the opposite side (right) there is more evenness of proportions. Some indices can be observed on the horizontal axis: 0 = species richness; 1 = Shannon index; 2 = Simpson's index; Inf = Berger-Parker index.

Hill's diversity profile demonstrated that parasite species richness was similar between 2020 and 2021 (0 on the q scale). However, the Shannon-Weaver index (1 on the q scale) was higher in 2021, suggesting that the increased importance of common species influenced the differences detected by the diversity index between years. The Simpson index (2 on the q scale) showed greater dominance in 2021. These results suggest that species richness was similar between the years, but the other indices that use abundance values showed that there was greater diversity in 2021 than in 2020 (Figure 6).

### Seasonal variation of metazoan parasites

The fish collected in the rainy season had a mean length of 8.3 ± 1.7 cm, and those collected in the dry season had a mean length of 7.1 ± 1.5 cm, which was a significant difference (t = 4.9091, p ≤ 0.0001) between these seasonal periods. The fish collected in the rainy season weighed 10.1 ± 6.7 g, while those collected in the dry season weighed 5.5 ± 4.0 g, which was also a significant difference (*U* = 1751.50; p ≤ 0.0001) between these seasonal periods. The relative condition factor of the hosts collected in the rainy season (Kn = 1.18 ± 1.58) and in the dry season (Kn = 1.07 ± 0.85) were significantly different (*U* = 2477.00; p = 0.002).

**Figure 7:**
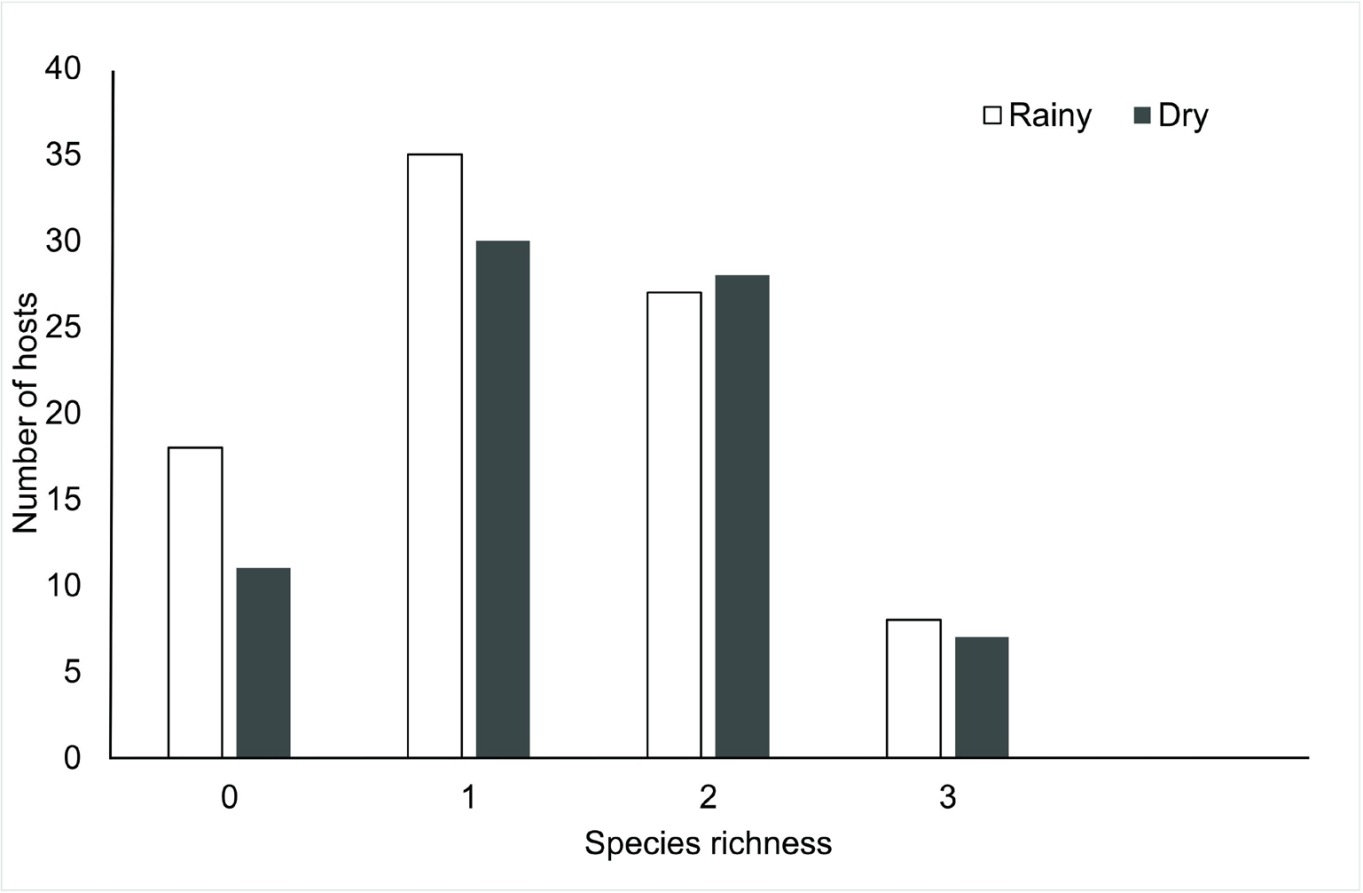
Species richness of metazoan parasites in *Eleotris pisonis* in the Amazon River during the rainy and dry seasons.

Totals of 495 parasites were collected in the rainy season and 383 in the dry season. In both the rainy and the dry season, hosts were predominantly infected by one parasite species (Figure 7). In the rainy season, 80.6% of the fish were parasitized; while in the dry season, 85.5% of the fish were parasitized. *Contracaecum* sp. was the dominant species in both seasonal periods. There was higher prevalence and abundance of *G. genarchella* in the dry season, while *Ergasilus* sp. had higher prevalence in the rainy season. The other species did not show any significant differences between seasonal periods (Table 5).

**Table 5:** Metazoan parasites in *Eleotris pisonis* in the Amazon River, eastern Amazon region, Brazil, collected during the rainy and dry seasons.

		**Rainy (n = 88)**			**Dry (n = 76)**				
**Parasite species**	**Infection sites**	**P (%)**	**MA ± SD**	**TNP**	**P (%)**	**MA ± SD**	**TNP**	**χ^2^**	***U***
*Contracaecum* sp. (larvae)	Intestine, stomach abdominal cavity and liver	69.3	3.6 ± 6.6	323	64.4	2.6 ± 3.7	203	0.43	3181.5
*Pseudoproleptus* sp. (larvae)	Intestine	0	0	0	1.3	0.01 ± 0.1	1	-	-
*Spirocamallanus inopinatus*	Abdominal cavity	0	0	0	1.3	0.01 ± 0.1	1	-	-
*Genarchella genarchella*	Pharynx, intestine, stomach, abdominal cavity and cecum pyloric	31.8	1.3 ± 2.8	119	63.1	2.3 ± 3.4	181	17.6^**^	2354.5**
*Neoechinorhynchus* sp.	Intestine and stomach	3.4	0.05 ± 0.2	4	0	0	0	-	-
Acarina gen. sp.	Gills	1.1	0.5 ± 1.9	1	0	0	0	-	-
*Ergasilus* sp.	Gills	22.7	0.01 ± 0.1	48	10.5	0.1 ± 0.3	9	5.9^*^	2912.0

P: prevalence; MA: mean abundance; SD: standard deviation; TNP: total number of parasites; χ^2^: chi-square test; *U*: Mann-Whitney test;

* p < 0.05;

** p < 0.001.

**Figure 8:**
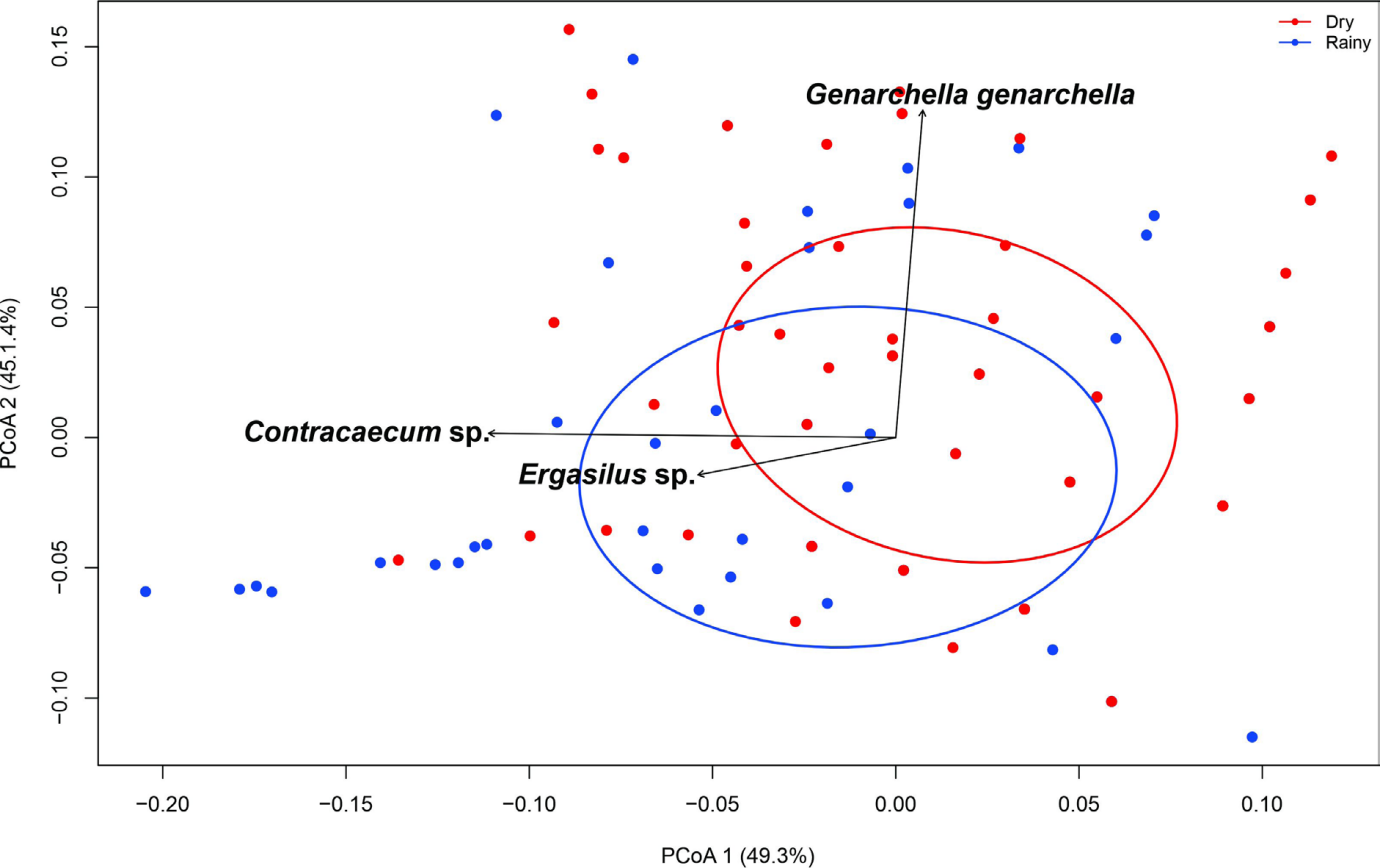
Principal coordinate analysis (PCoA) using a Bray-Curtis distance matrix for communities of metazoan parasites of *Eleotris pisonis* in the Amazon River, eastern Amazon region, Brazil, during the rainy and dry seasons. The percentage of the variation explained by the principal coordinates plotted is indicated on the axes.

Axes 1 and 2 of the PCoA results (Figure 8) were responsible for 94.3% of the total variation in the composition of parasite abundance in the rainy and dry seasons. Despite the overlap due to sharing of some species, the PCoA showed that there were significant differences (PERMANOVA: F = 6.6655; p ≤ 0.003) in the parasite infracommunities between the seasonal periods, mainly influenced by variations in the abundance of *G. genarchella* (R^2^ = 0.7782; p ≤ 0.001), larvae of *Contracaecum* sp. (R^2^ = 0.6065; p ≤ 0.001) and *Ergasilus* sp. (R^2^ = 0.1533; p ≤ 0.001) (Figure 8).

**Figure 9:**
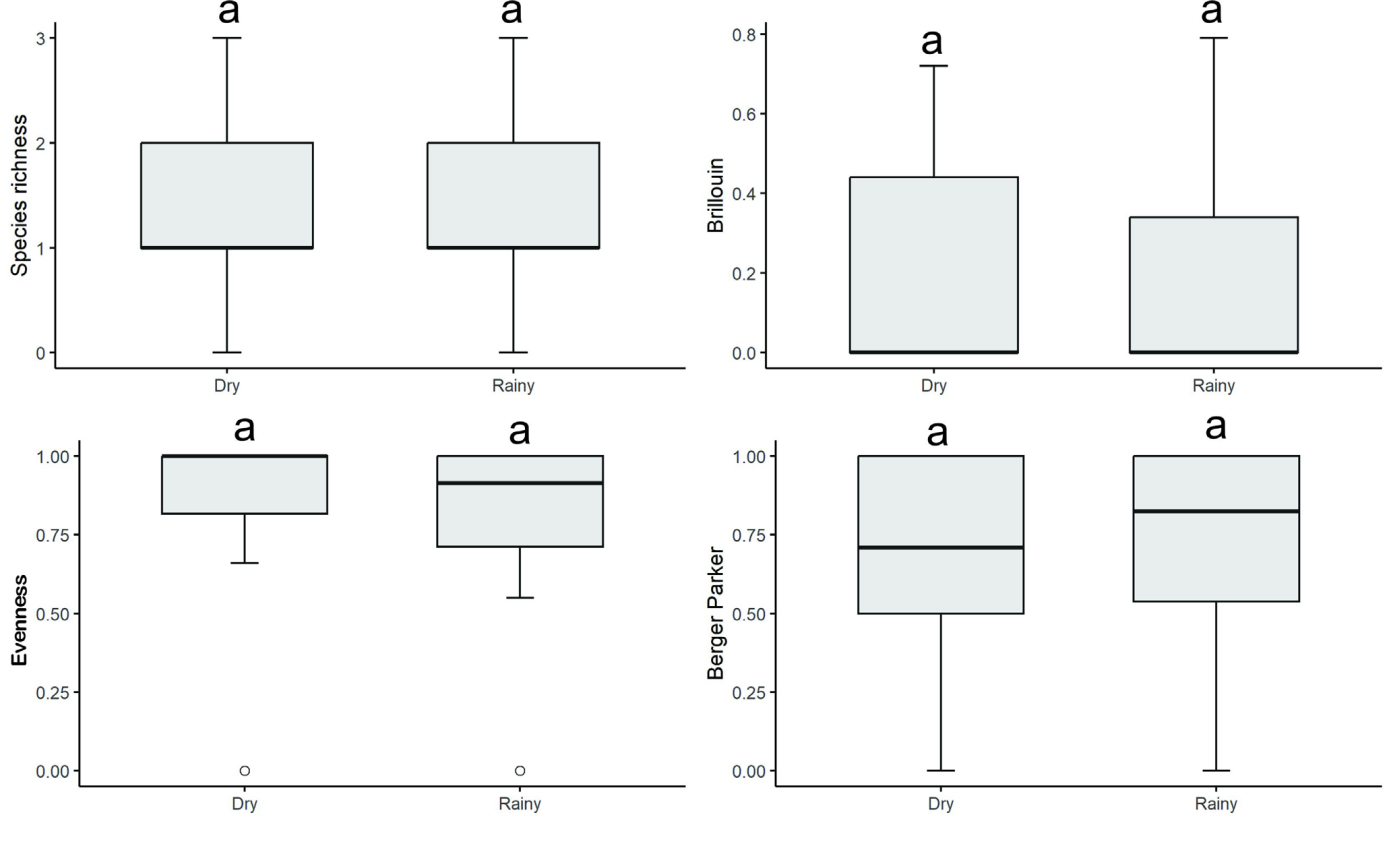
Diversity parameters of metazoan parasites in *Eleotris pisonis* in the Amazon River, eastern Amazon region, Brazil, during the rainy and dry seasons. (Box plots represent medians, interquartile ranges, minimum–maximum ranges and outliers). Different letters indicate differences between the medians according to Dunn's test (p < 0.001).

**Figure 10:**
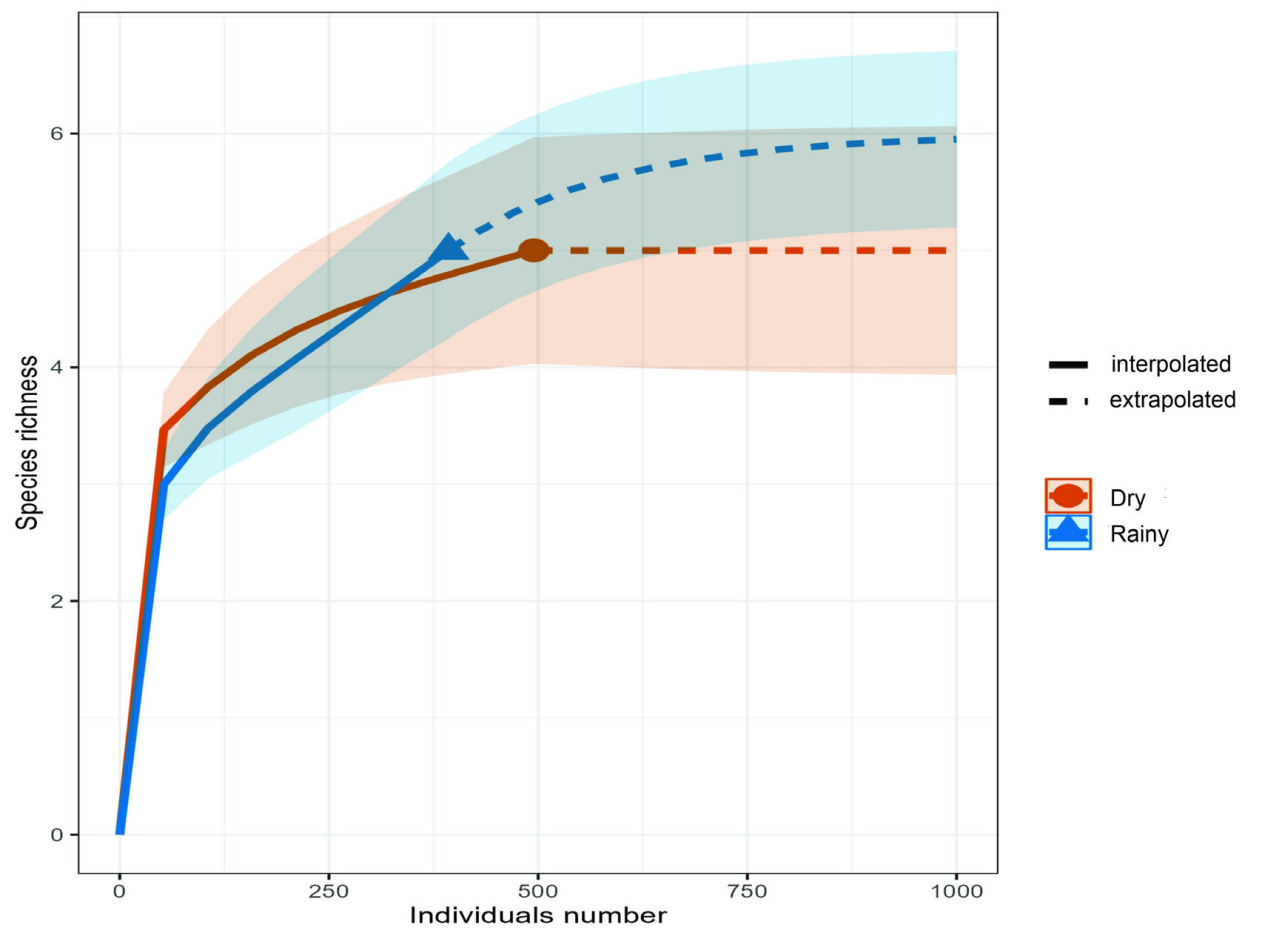
Species accumulation curve for metazoan parasites in *Eleotris pisonis* in the Amazon River, eastern Amazon region, Brazil, during the rainy and dry seasons.

Parasite species richness (χ^2^ = 0.8869, p = 0.346), Brillouin diversity index (χ^2^ = -1.27, p = 0.203), evenness (χ^2^ = -1.76, p = 0.0778) and Berger-Parker dominance (χ^2^ = 0.424, p = 0.672) did not show any significant differences between seasonal periods (Figure 9). The parasite species accumulation curve did not show any differences in species richness between the seasonal periods. This showed that the representativeness of the species collected, regarding parasite richness, was not affected by the sampling effort between the seasonal periods, given that the two curves tended towards stability (Figure 10).

**Figure 11:**
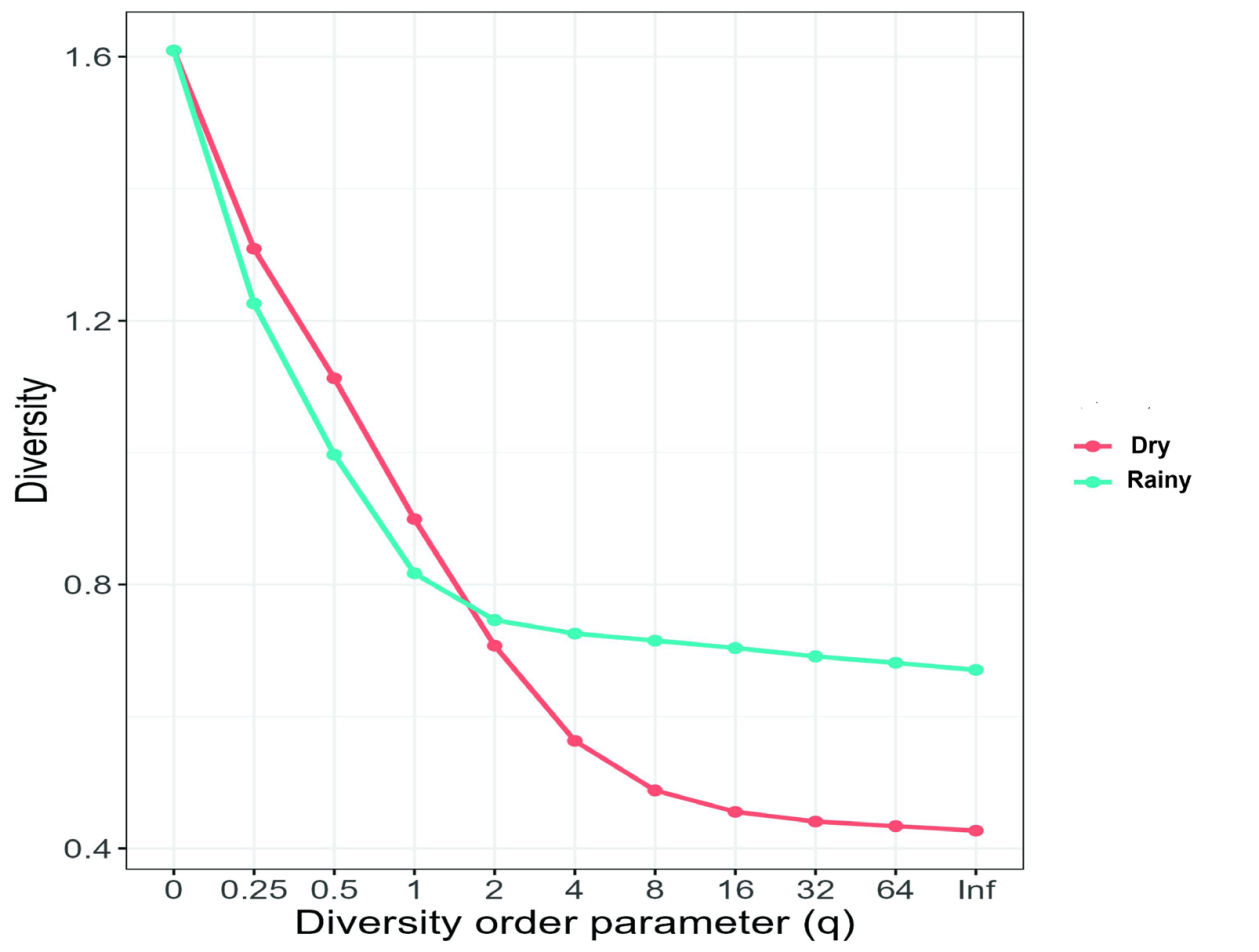
Hill diversity profile for parasite diversity in the rainy and dry seasons in *Eleotris pisonis*. On the horizontal axis (left), rare species become more important, while on the opposite side (right) there is more evenness of proportions. Some indices can be observed on the horizontal axis: 0 = species richness; 1 = Shannon index; 2 = Simpson's index; Inf = Berger-Parker index.

Hill's diversity profile showed that parasite species richness did not differ between the seasons (rainy and dry) (0 on the q scale). However, the Shannon-Weaver index (1 on the q scale) for the dry season was slightly higher, suggesting that the increased importance of common species influenced the differences detected by the diversity index between seasonal periods. On the other hand, the Simpson index (2 on the q scale) for dominance was higher in the rainy season. These results suggest that species richness was also similar between seasonal periods. However, the other indices that use abundance values alternated between seasonal periods (Figure 11).

## Discussion

### Component communities of metazoan parasites

The component community of metazoan parasites in *E. pisonis* in the Amazon River comprised three species of Nematoda, one Digenea, one Acanthocephala, one Crustacea and one Arachnida. However, in *E. pisonis* collected from the Guadeloupe islands in the Caribbean, only *Cucullanus caballeroi* Petter, 1976, was found (Petter et al., 1977); in hosts from the mouth of Keelung River, Taiwan, the acanthocephalans *Brentisentis uncinus* Leotta et al., 1982 and *Gorgorhynchus satoi* Morisita, 1937, were found (Leotta et al., 1982); and in *E. pisonis* in the Matapi River, a tributary of the Amazon River, no crustacean parasite was found (Neves & Tavares-Dias, 2019). These differences in the component communities and richness of host species can be attributed to differences in the environment, diet, seasonal period and sampling effort. Regarding sampling effort, the number of specimens of *E. pisonis* sampled in the present study was higher than in the previous studies cited above. In addition, new records of metazoan parasites for *E. pisonis* are reported in the present study.

The presence of these endoparasites in *E. pisonis* may be related to its carnivorous feeding habit (Froese & Pauly, 2023), since infections by endoparasites have mainly been related to the diet of the host fish (Oliveira et al., 2017; Negreiros et al., 2019a, b; Lima et al., 2021, 2022, 2023). However, infection by the ectoparasite *Ergasilus* sp. may be related to the specificity of the parasite and the reproductive period, as observed in *Colomesus asellus* Thatcher & Boeger, 1983, from the Môa River, in the eastern Brazilian Amazon region (Virgilio et al., 2021) and from the Amazon River (Lima et al., 2023). Infections by mites may be associated with environmental conditions, host stress levels and accidental infections in the environment (Olmeda et al., 2011; Lizama et al., 2013).

Highly aggregated dispersion patterns were found for *Contracaecum* sp., *G. genarchella* and *Ergasilus* sp. in *E. pisonis*. These distribution patterns are common among freshwater fish species living in different natural environments (Tavares-Dias & Neves, 2017; Oliveira et al., 2017; Neves et al., 2021). Aggregated dispersion has been linked to the genetic variability of the host population, decreased interspecific competition between parasites, decreased damage to the host and environmental factors (Poulin, 2011; Tavares-Dias & Oliveira, 2017).

The presence of larvae of *Contracaecum* sp. and *Pseudoproleptus* sp. was an indication that *E. pisonis* is an intermediate host due to its carnivorous feeding habit (Bartolette et al., 2018; Froese & Pauly, 2023). Shrimps, gastropods and crabs were found in the stomach of *E. pisonis* (personal observation), thus indicating that this host occupies a superior position in the food web. Larvae of *Contracaecum* sp. were the dominant species, presenting a higher level of infection than that of other nematodes such as *Pseudoproleptus* sp. and *S. inopinatus*. Infection by *Contracaecum* sp. was also reported in *Pimelodus ornatus* Kner, 1958 (Lima et al., 2021, 2022) and *C. asellus* (Lima et al., 2023), collected in the same study area from which the specimens of *E. pisonis* were collected. However, there have been reports of occurrence of *Contracaecum* sp. in other species of Amazonian fish such as *Hemibrycon surinamensis* Géry, 1962 (Hoshino et al., 2014), *Metynnis lippincottianus* Cope, 1879 (Hoshino & Tavares-Dias, 2014), *Astronotus ocellatus* Agassiz, 1831 (Tavares-Dias & Neves, 2017) and *Astronotus crassipinnis* Heckel, 1840 (Santos et al., 2018).

*Spirocamalanus inopinatus* is a nematode with wide geographic distribution and with records in different fish species in Brazil (Neves et al., 2020), but the present study provided the first record of this nematode in *E. pisonis.* However, the infection levels in *E. pisonis* were low in comparison with those reported in *Pimelodus blochii* Valenciennes 1840 in the Iaco and Acre Rivers (Negreiros et al., 2018, 2019b), *Pimelodus maculatus* Lacepéde, 1803, in the Guandu River (Albuquerque et al., 2008) and *P. ornatus* in the Amazon River (Lima et al., 2021). These findings demonstrate that *S. inopinatus* is a common nematode in Amazonian fish, but with low levels of infection in *E. pisonis*.

Larvae of *Pseudoproleptus* sp. were found in *E. pisonis*, and this paratenic nematode has also been well documented in several species of Amazonian fish (Melo et al., 2011; Tavares-Dias et al., 2014; Oliveira et al., 2018; Souza et al., 2020) and in the Amazonian shrimp (*Macrobrachium amazonicum* Heller, 1862) and some aquatic insects that are used as intermediate hosts (Moravec & Santos, 2009). However, some fish species are used as definitive hosts for this nematode, such as *Hoplias malabaricus* Bloch, 1794, which feeds on the cichlids *Satanoperca jurupari* Heckel, 1840, and *Aequidens tetramerus* Heckel, 1840. In turn, these are intermediate hosts for the larvae of *Pseudoproleptus* sp. (Melo et al., 2011). This pattern can also be found in *E. pisonis*, given that we found an individual of *E. pisonis* feeding on another specimen of this species. Occurrence of cannibalism among *E. pisonis* has previously been documented for this species, with low occurrence (0.6%) (Perrone & Vieira, 1991). Nonetheless, the possibility that *E. pisonis* also occupies higher levels in the food web due to its infrequent cannibalistic habit cannot be ruled out.

In *E. pisonis*, the presence of *G. genarchella* and *Neoechinorhynchu*s sp. indicated that this fish is a definitive host for these endoparasites (Cardoso et al., 2017; Ferrari-Hoeinghaus et al., 2007). Mites presented low levels of infection in the gills of *E. pisonis* in the Amazon River, but have been widely found on a variety of vertebrates, including fish and piscivorous birds (Lizama et al., 2013). In Amazonian fish, mite infection has been reported in *Colossoma macropomum* Cuvier, 1816 (Gonçalves et al., 2018), *Brachyplatystoma vaillantii* Valenciennes, 1840 (Brito-Junior & Tavares-Dias, 2021) and *Hemiodus unimaculatus* Bloch, 1794 (Almeida et al., 2021).

*Ergasilus* sp. are generally ectoparasites with a certain degree of host specificity. Their level of occurrence in *E. pisonis* was lower than that of *C. asellus* (Lima et al., 2023). Ergasilid species have wide distribution in the Amazon River system, with occurrence in several Amazonian fish species (Thatcher & Boeger, 1983; Vasconcelos & Tavares-Dias, 2016; Borges et al., 2018; Sousa et al., 2019; Lima et al., 2023); however, it was recorded for the first time in *E. pisonis* in the present study.

### Annual variation in communities and infracommunities of metazoan parasites

The specimens of *E. pisonis* collected in 2020 were larger and heavier, and had a better relative condition factor than those collected in 2021. Despite these differences in *E. pisonis* populations, five species of parasites were found in both years, with predominance of larvae of *Contracaecum* sp. This may have been related to the low specificity and high reproductive and infection rates of this generalist nematode (Neves et al., 2013). Although the body size of the hosts is one of the determining factors for the diversity, richness and abundance of parasites (Marcogliese et al., 2016; Baia et al., 2018), such differences in *E. pisonis* may also be related to the spawning peaks of this host fish species after the Amazonian floods. These factors would cause a difference in diet between juveniles and adults, and would show that sexual maturation is likely to be a factor influencing the trophic ontogeny of the species. This would cause a decrease in intraspecific competition (Perrone & Vieira, 1991), thus generating better feeding and reproduction conditions.

Species richness of parasites and Brillouin's diversity were higher in 2021. Similar findings were reported for *P. ornatus* and *C. asellus* collected from the Amazon River in 2020 and 2021 (Lima et al., 2022, 2023). Such differences may have been influenced by variations in physicochemical characteristics of these environments.

For *E. pisonis*, the differences in the parasite infracommunities between the years 2020 and 2021 indicated in the PCoA were mainly due to the abundance of *Contracaecum* sp., *G. genarchella* and *Ergasilus* sp. Similar results were reported by Lima et al. (2023) in *C. asellus* collected from the Amazon River. This indicates that such differences may be related to the availability of intermediate hosts of the endoparasites in the environment, and to the reproductive period of the ergasilids (Villalba‐Vasquez et al., 2018; Hoshino & Tavares-Dias, 2019; Lima et al., 2022, 2023). In addition, the levels of *G. genarchella* infection were higher in 2021, and this variation may have been more related to seasonal variation than to any influence of the availability of infective stages of these digeneans in the environment. In *P. ornatus* (Lima et al., 2022) and *C. asellus* (Lima et al., 2023) in the Amazon River, short-term annual variations in parasite communities and infracommunities have also been correlated with the seasonal cycle (rainy/dry), availability of infectious stages, changes to the parasite species recruitment process, urban eutrophication and host body size.

*Genarchella genarchella* and larvae of C*ontracaecum* sp. were present in both of the years studied, thus indicating that contact between *E. pisonis* and the infective forms of these endoparasites did not vary between these two years. Similar findings were reported by Hoshino et al. (2014) in *H. surinamensis* collected from a tributary of the Amazon River, in which the levels of infection by *G. genarchella* and larvae of *Contracaecum* sp. were stable among the years studied, as also were the levels in *C. asellus* in the Amazon River (Lima et al., 2023).

In *E. pisonis*, infestations by *Ergasilus* sp. were observed in both years of the present study. In *C. asellus*, infestation by *E. colomesus* also occurred in both years studied, due to segregation of the hosts by size for feeding (Lima et al., 2023). The absence of segregation by size for feeding and reproduction among *E. pisonis* may have facilitated encounters with these ergasilids during the years studied, and may have facilitated their attachment to the hosts’ gills for reproduction (Williams & Bunkley-Williams, 2019).

Mites were observed on the gills of *E. pisonis* only in 2020. These parasites are usually found on the gills, integuments and digestive tracts of their host fish (Olmeda et al., 2011; Lizama et al., 2013). However, some authors have considered them to be unusual parasites in fish, and others have taken the view that mites are not fish parasites, since the habitats and behavior of fish do not contribute to the infestations found (Olmeda et al., 2011; Lizama et al., 2013; Brito-Junior & Tavares-Dias, 2021). However, mites can proliferate and infect weak or stressed fish, under certain environmental conditions, thereby causing serious damage to the host. In fish in Australia, Europe and North America, some genera of mites have been isolated and correlated with high host mortality (Olmeda et al., 2011; Lizama et al., 2013).

### Seasonal variation of communities and infracommunities of metazoan parasites

Specimens of *E. pisonis* collected in the rainy season were larger and heavier and thus presented a better relative condition factor. This result indicated that the fish were feeding better during this seasonal period and/or were in the reproductive period, as indicated by some individuals in which mature gonads were found. Such observations were also reported by Perrone & Vieira (1990) in *E. pisonis* collected from the estuarine region of the Jucu River, in the State of Espírito Santo, Brazil, where females with mature ovaries occurred frequently from February to June and, specifically, soon after the river flood peak. This result emphasized in rivers, such that changes in water volume caused by seasonal changes directly affected the existing community, influencing changes mainly with regard to the feeding, reproduction and sizes of fish populations (Lowe-McConnell, 1967). In the Amazon basin, the rainy and dry seasons generally influence the communities of invertebrates and fish that serve as food for many carnivorous fish such as *E. pisonis*. In the rainy season, there is greater diversity of the zooplankton and other invertebrates that form part of the diet of these fish, thus improving their body condition (Gonçalves et al., 2016; Tavares-Dias et al., 2014).

Populations of *E. pisonis* do not show spatial segregation between adults and juveniles. However, there is a difference in feeding habits between juveniles and adults, caused by sexual maturation, which influences the trophic ontogeny of this species (Perrone & Vieira, 1990). Thus, adult individuals (larger and heavier) reproduce during the rainy season, while occupying the same space as young individuals, at a time when conditions for development are better. Spawning peaks occurred in the dry season, at a time when young individuals (smaller and less heavy) with less favorable feeding conditions are found. This corroborated the fact that smaller and less heavy individuals were found in the dry season.

In *E. pisonis*, parasite species richness (five species in each seasonal period), diversity, evenness and Berger-Parker dominance index did not differ between seasonal periods. In addition, *Contracaecum* sp. was the dominant species in both seasonal periods. Similar results were reported in relation to *P. blochii* in the Acre River, where these diversity parameters were not influenced by seasonality (Cavalcante et al., 2020). However, mites and *Neoechinorhynchus* sp. occurred only in the rainy season, while *Pseudoproleptus* sp. and *S. inopinatus* occurred only during the dry season. Among the possible influences on the seasonal pattern found in our data, the absence of a spatial seasonal segregation pattern (Perrone & Vieira, 1990) and the abundance of hosts in both seasons can be cited.

The PCoA showed seasonal differences in the infracommunities of *Contracaecum* sp., *G. genarchella* and *Ergasilus* sp. in *E. pisonis*. The period of intense rainfall (rainy season) and the less rainy period (dry season) in the Amazon region are well defined. These seasonal variations may be responsible for variations in parasite species recruitment, food availability for hosts and, consequently, infective stages in the environment (Neves et al., 2013; Gonçalves et al., 2016; Hoshino & Tavares-Dias, 2020). They may also influence the reproductive period of Amazonian fish (Cavalcante et al., 2020). These variations can alter the habitats of fish populations and water velocity, thus increasing the stress levels among host fish and their susceptibility to parasitic infections and, consequently, altering the structure of parasite communities and infracommunities in fish host populations (Gonçalves et al., 2016).

In *E. pisonis*, G. *genarchella* was found in both seasonal periods, but the highest levels of infection were observed in the dry season. The infective stages of this digenean are present during both seasonal periods (rainy and dry), but more frequently in the dry season. In *C. asellus* in the Amazon River, *G. genarchella* infection also occurred in both seasons (Lima et al., 2023). In contrast, for *P. ornatus* in the Amazon River, infection by this digenean only occurred in the rainy season (Lima et al., 2022), when the chances of host fish encountering the infective stages of this digenean are greater.

In *E. pisonis*, the presence of *Ergasilus* sp. was observed only in the rainy season, as also were *Telotha henselli* Von Martens, 1869, in *P. ornatus* (Lima et al., 2022) and *Argulus pestifer* Ringuelet, 1948, in *C. asellus* (Lima et al., 2023). On the other hand, in *C. asellus*, infestation by *E. colomesus* occurred in both seasons, but with higher levels occurring in the dry season. Females of the genus *Ergasilus* attach themselves to the gills and remain there until their eggs are mature, after which they detach from their hosts and release the eggs into the environment (Williams & Bunkley-Williams, 2019). Our results indicate, therefore, that the ergasilid species found in *E. pisonis* in the Amazon River presents higher reproduction levels during the rainy season.

## Conclusions

About 59.1% of the parasites were larvae, thus indicating that *E. pisonis* is an intermediate or paratenic host. Our results, based on sampling over two years and in both seasons (rainy and dry), indicated that the parasites differed between the years and seasons regarding the diversity of some infracommunities. The little effects of annual and seasonal variations on the diversity and levels of infection were related to variations in rainfall levels and, consequently, to the availability of infective stages of parasites with direct and indirect life cycles and changes in the recruitment of parasite species in the environment caused by seasonality. Hence, the results do not corroborate the hypothesis that seasonal cycle (rainy/dry) would not influence the communities of parasites. Furthermore, this study was the first to investigate the effects of annual and seasonal variations of metazoan parasites on *E. pisonis*. Consequently, this study provides the first record of occurrences of larvae of *Contracaecum* sp. and *Pseudoproleptus* sp., and adult individuals of *S. inopinatus*, *G. genarchella*, *Neoechinorhynchu*s sp., mites and *Ergasilus* sp., in *E. pisonis*.
